# Modelling capability factors of logistics industry based on ISM-MICMAC

**DOI:** 10.1016/j.heliyon.2024.e40539

**Published:** 2024-11-19

**Authors:** Mohammad Kamrul Hasan, Xunping Lei, Arbia Hlali, Zixiang Bian

**Affiliations:** a School of Business Administration, Tongling University, Tongling, Anhui, 244061, China; b Department of Management, Taibah University, Saudi Arabia; c School of Communications and Transportation, Shijiazhuang Tiedao University, Shijiazhuang, Hebei, 050043, China

**Keywords:** Logistics capability factors, Structural modelling, ISM-MICMAC, Performance

## Abstract

Supply chain disruptions and global pandemics have revealed vulnerabilities within the logistics industry in recent years. Consequently, there has been a growing emphasis across various industries on enhancing logistics capabilities. The focus on improving logistics performance through capability factors has shifted the paradigm toward prioritizing these factors. In light of this transition, this study develops a structural model to identify the logistics capability factors (LCFs) that contribute to logistics performance. To define the interrelationships and interdependencies among LCFs, expert-based interpretive structural modeling (ISM) combined with cross-impact matrix multiplication applied to classification (MICMAC) is used. A complex ISM model and a MICMAC diagram, based on driving and dependency power, are constructed to determine and categorize LCFs. The findings indicate that the demand management interface (DMI) of service capability is a pivotal factor in enhancing logistics capability, followed by the significance of information management and technological innovation capabilities. The results of this study have several implications for understanding the relationships among LCFs and for extending knowledge through the application of the ISM-MICMAC technique. Both theoretical and managerial insights are highlighted, providing guidance for supply chain leaders and operational managers.

## Highlights

• The capability factors are crucial for enhancing logistics performance.
• ISM-MICMAC is utilized to assess the interrelationships among the capability factors.
• The most influential relationships among the major capability factors have been identified.
• Supply chain managers can identify the key capability factors, analyze them, and enhance logistics capabilities.

## 1 Introduction

Global logistics have experienced significant disruptions and disorder over the past decade. Recent challenges, including global pandemics and epidemics [1], geopolitical instability, and environmental issues, have exposed the vulnerabilities inherent in the logistics industry. Consequently, the current paradigm shift in logistics performance is characterized by an explosion of product modulation, service innovation, capability enhancement, and information digitalization, which present substantial challenges to operational effectiveness [2,3]. Furthermore, the global energy crisis and a multitude of supply chain (SC) risk factors have tested the resilience of logistics industries. It introduced unprecedented complexity, and disrupted the equilibrium of supply and demand networks. Stakeholders in the logistics industry and those providing related services have faced intricate challenges in supply chain management (SCM) activities. Therefore, it is essential to diversify the logistics capabilities within the industry in response to the evolving patterns of SC behavior [4]. A number of strategies have been proposed in the literature, including studies on SC resilience, agility, flexibility, viability, and dynamic innovation. However, very few studies have attempted to examine logistics capability factors (LCFs) in a structured manner. In this context, further efforts are necessary to enhance logistics capabilities, efficiency, and operational flexibility [5]. Thus, understanding LCFs is essential for organizations to achieve unparalleled levels of capability and effectiveness [6].

The anticipated normalization of logistics capabilities in 2023 was predicted based on the sector's strong fundamentals, which are expected to ensure resilience in the face of challenges [4]. A systematic approach to learning and adapting to challenging circumstances has enabled efficient operations to adopt the best business practices in the industry. This is facilitated by logistics benchmarks and dynamic innovation. Logistics benchmarking-including internal, competitive, and comparative benchmarking-serves as an operational strategy for the logistics industry to improve performance by identifying weaknesses [7]. This process provides a rationale for strengthening internal capabilities [8]. In addition to benchmarks, the capability for innovation within firms represents a critical dynamic capability for navigating the rapidly evolving business environment, which is often characterized by unpredictable disruptions [9]. It is therefore essential for logistics firms and SC members to embrace innovative solutions and implement agility to ensure optimal performance [9]. Dynamic innovation capabilities are recognized as pivotal drivers for navigating volatile business environments [9,10]. It encompasses the development of distinctive services and technological advancements. Such capabilities promote innovation-oriented strategies that improve the resilience of companies and the efficiency of logistics service providers (LSPs) [11].

In addition, the expansion of SC networks has compelled LSPs to prioritize logistics practices and service capabilities [12]. The logistics service capability, particularly through flexible practices, collaborates with key suppliers and customers effectively to respond to uncertainties and unexpected circumstances [13]. So, the dimensions of SC flexibility—internal, supplier, and customer flexibility—are of paramount importance in addressing crises and synchronizing partners in order to enhance resilience [14]. The enhancement of LCFs and the establishment of SC synchronization among flexibility, resilience, agility, and the Internet of Things (IoT) are emphasized in previous studies. In a study by Agrawal et al. [14], the authors empirically tested the hypothesis that a flexible SC can improve both resilience and robustness. It ultimately leads to enhanced overall SC performance. The multi-dimensional logistics capability of flexibility can exert a positive influence on customer responsiveness through dynamic innovation within a firm [15]. In addition to flexibility, technological innovation can impact service time and draw the attention of new business partners [3]. So, it would be practical for the logistics industry to further explore their technological capabilities, particularly before making significant investments in SC risk management [16].

Moreover, the collaborative strategies and information-based capabilities among logistics firms are assumed to enhance a firm's performance and improve management functions [17]. Information-based capabilities, such as information sharing, have the potential to simultaneously enhance distribution performance and service efficiency and reduce costs [18]. Logistics service capability, a demand-oriented or customer-oriented approach [7], is defined as the capacity to respond effectively to market dynamics [19]. It emerges from four key interfaces: the demand-management interface (customer service and logistics quality), the supply-management interface (low-cost distribution and low-cost supply), the information-management interface (information sharing and information technology), and the coordination capabilities (both internal and external) [7]. The potential associations and interrelationships among these capabilities have remained largely unexplored thus far. The factors contributing to capability are crucial for effective SCM. There has been limited attention in the existing literature to explore the complex framework of these capability factors through a structural model. In particular, we argue that modeling LCFs provides valuable insights for LSPs and the manufacturing sector. Thus, it gives potential motivations to conduct this study.

Although previous studies have acknowledged the significant effects of various capability factors, the domain has largely overlooked the structural relationships among them. A primary limitation of existing research is its focus solely on the application of capability factors to enhance SC performance without delving deeper into the underlying trends. This gap provides a basis for further exploration and modeling of LCFs. It offers both theoretical and managerial insights. A few scholars have already studied the factors influencing logistics capability. Rajesh [16], among others, attempted to model firms’ technological capabilities and SC resilience. Hashemi Petrudi [5] developed a structural model for supply chain viability factors. Zhong et al. [20] modeled SC resilience capability factors in agri-food. Sindhwani and Malhotra [21] examined agile manufacturing systems. Mangla et al. [22] studied factors influencing sustainable risk in the SCM. But no coordinated effort has yet been made to identify and rank LCFs or establish a structural relationship among them using interpretive structural modelling (ISM). It creates a gap in the existing literature. Thus, a structural model that clarifies the interrelationships and interdependencies among LCFs is attempted through this study.

To address this gap, this study contributes in several aspects. First, our research advances knowledge in the field of logistics capability by identifying and ranking LCFs and developing a structural model. Second, it investigates the potential connections among LCFs based on their interrelationships and interdependencies that influence performance. Third, the proposed framework provides a graphical analysis of the intricate interrelationships among these capability factors, offering a simplified representation of their prioritization. This representation equips managers with a framework for understanding these interrelationships, thereby facilitating informed decision-making within their organizations. The findings will have both direct and indirect effects on companies’ SC capability. It will further assist managers in enhancing their capability factors over their competitors. Thus, this study aims to bridge the gap and highlights the research objectives through the following research questions.

RO1 What are the critical logistics capability factors?

RO2 How are these factors ranked based on their driving and dependency power?

RO3 How do the factors interrelate each other?

RO4 How can organizations formulate management strategies to enhance their logistics capability?

The remainder of this paper is organized as follows. Section 2 reviews the relevant literature to provide a theoretical background and define key LCFs. Section 3 outlines the methodology and analyzes the results. Section 4 discusses the findings and their implications. Finally, Section 5 synthesizes the study's concluding remarks and highlights future prospects.

## 2 Theoretical background and literature review

The theoretical framework commences with an extensive review of existing literature on LCFs, followed by a concise overview of the factors influencing logistics capability in the subsequent section.

The exploration and classification of logistics capabilities are integral to SCM. Numerous researchers have explored the influence of various capability factors and their applications in improving logistics performance. For instance, Bagheri et al. [23] identified key technological capabilities in SC sustainability using an ISM approach and demonstrated information technology as the most significant driver of other technological capabilities in the industry. In their study, Chen et al. [24] concentrated on the emergency logistics response capabilities of public emergencies. They found that logistics response capability varies among Chinese regions on different scales. In addition, Sun et al. [25] applied a combination of clustering techniques and fuzzy matter element analysis to extract the logistics capabilities of key cities along China's "Belt and Road" initiative. They identified various LCFs as essential for the circulation of resources and the economic clustering of cities within the "Silk Road Economic Zone. Wang et al. [26] highlighted the importance of logistics innovation capabilities in mitigating SC risks and illustrated that organizations can effectively counter the negative impacts of risks by enhancing their logistics innovation capabilities and strengthening resilience. Interconnections among logistics resource, capability, and innovation have been established by Yang et al. [27], Puspitasari and Kusumawardhani [28], and Yang et al. [29]. However, Zhao et al. [30] explained the pros and cons of logistics service capabilities that focus on customer engagement and information management to demonstrate organizational performance. A summary of the current literature is presented in Table 1.

**Table 1:** Summary of previous studies.

NO.	Author(s)	Focus	Methodology	Findings/contributions
**1**	Agrawal et al. [14]	The influence of SC flexibility on coordination, resilience, and robustness.	Cross sectional approach	Emphasized the robust predictive nature of SC flexibility
**2**	Arabelen and Kaya [12]	Evaluated of logistics service quality dimensions.	Qualitative approach	Five dimensions of logistics service quality and 24 associated factors have been identified as guidelines for quality management practices in logistics applications and transportation.
**3**	Bagheri et al. [23]	Technological capabilities in SC sustainability.	ISM-MICMAC method	Information technology has been the most significant driver of technological capabilities in companies' SC sustainability.
**4**	Cheng et al. [31]	Digital capability and green innovation for green SC collaboration	Structural path analysis	Digital capabilities can significantly enhance the performance of green innovation in manufacturing companies, thereby promoting the growth of sustainable practices through technological advancements.
**5**	Chen et al. [24]	Emergency logistics response capabilities	Entropy weight TOPSIS	Concentrated on the emergency logistics response capabilities during public emergencies. The researchers found that the logistics response capabilities vary across different regions of China and on various scales.
**6**	Chowdhury et al. [1]	Flexibility in enhancing SC resilience	Multi-method and multi-study approach with fsQCA	Implementing resilience strategies alone is ineffective in improving SC performance, while resilience strategies combined with the nullification of risk factors enhance SC performance.
**7**	Evangelista et al. [49]	Knowledge-based human resource management (HRM) and organizational performance	PLS-SEM	Provides an understanding of the role of HRM and logistics capability in the performance of small logistics service companies.
**8**	Fanghu et al. [50]	Logistics capability evaluation index system	Entropy weight TOPSIS	The regional logistics capacity shows a trend of increasing year by year, but the logistics capacity of different provinces within the region has a large room for improvement.
**9**	Gligor and Holcomb [7]	logistics capabilities and their impacts on firms' performance	SEM	The integration of logistics capabilities significantly impacts both the operational and relational performance of a firm. It has the potential to reduce overall costs while fostering better relationships with customers and delivering superior customer value.
**10**	Hashemi Petrudi et al. [5]	SC viability factors	Modified Total ISM	Digital engagement has the most significant impact on SC viability, followed by energy and resource consumption, as well as job safety and labor health.
**11**	Huang and Huang [10]	Logistics capabilities scale for LSPs	Theoretical approach and survey questionnaire	Theoretically proposes 24 key logistics capabilities for LSPs drawn from previous studies and interviews with logistics experts and executives.
**12**	Humdan et al. [9]	Logistics innovativeness and SC agility in the service industry	SEM and fuzzy set qualitative comparative analysis (fsQCA)	SC agility significantly mediates the relationship between innovativeness and logistics performance, providing a more accurate prediction of performance outcomes.
**13**	Jafari et al. [15]	Effects of SC flexibility on customer responsiveness	Hierarchical linear regression and moderation analyses	SC flexibility, as a multi-dimensional construct, affects customer responsiveness.
**14**	Piprani et al. [32]	The effect of multi-dimensional supply chain flexibility (MDSCF) in improving resilience	SEM	The findings indicated that MDSCF significantly contributes to improving SC resilience and contributed.
**15**	Shang and Marlow [18]	The interrelationship between logistics capabilities, logistics performance, and financial performance.	SEM	Information-based capability is the most critical factor, as it significantly influences benchmarking capability, flexibility, and logistics performance.
**16**	Sun et al. [25]	Measurement of logistics capability	Fuzzy matter-element analysis	Logistics capacity of cities along the 21st Century Sea Silk Road is more robust than that of other cities.
**17**	Zhong et al. [20]	SC resilience capability factors in agri-food supply chain	DEMATEL and ISM-MICMAC	SC leadership is a fundamental factor for fostering agri-food supply chain resilience.

Sinkovics and Roath [19] coordinated the logistics performance of manufacturers through strategic orientation, operational flexibility, and SC collaboration. They demonstrated that capabilities may impact performance if efforts are focused on developing the “right” capabilities and performance. Cheng et al. [31] investigated the collaboration between digital capability and green innovation from a green supply chain (GSC) perspective and stated that digital capability significantly and favorably impacts green innovation and SC collaboration. Shang and Marlow [18] used structural equation modeling (SEM) to build relationships among logistics capabilities, logistics performance, and financial performance in Taiwan's major manufacturing firms. This study demonstrated that information-based capability is very critical for benchmarking and flexibility capabilities to stimulate logistics performance. Piprani et al. [32] modeled SC flexibility and resilience using Partial Least Squares-SEM (PLS-SEM) and indicated that multidimensional SC flexibility significantly contributes to improving resilience. Sabahi and Parast [33] studied firms' resilience using the dynamic capability view to examine the relationship between firms' innovation capability and response to disruptions. Dovbischuk [11] showed that firms' dynamic innovation-oriented capability enhances service providers' performance capability. Thus, innovation and service diversification in the logistics industry positively influence logistics performance [34]. Furthermore, Bagchi [6], Bagchi [8], Shang [35], and Daugherty et al. [36] have synthesized the capabilities required for efficient performance in logistics, identifying benchmarking, innovation, and information capability as being of particular importance.

In addition, Gligor and Holcomb [37] investigated the role of logistics capabilities in achieving SC agility. In a study involving 387 enterprises in Portugal, Ferreira et al. [38] demonstrated that dynamic innovation capabilities significantly impact competitive advantage. Gligor and Holcomb [7] defined integrated logistics capabilities as encompassing logistics coordination, cooperation, and internal communication for operational excellence. They illustrated how firms can develop these integrated logistics capabilities and how such capabilities influence firms' performance. Higginson and Alam [39] emphasized the importance of information-sharing capabilities among SC members. Other researchers, including Kirono et al. [17], Moberg et al. [40], Gligor and Holcomb [37], Lim et al. [41], Shang and Marlow [18], and Zhao et al. [30], underscored the significance of information technology, quality information, and commitment to advance information technology in achieving operational excellence. Huang and Huang [10] blended logistics flexibility capabilities with innovation and service capabilities to provide enterprises with a competitive advantage. Ilmudeen et al. [42] identified a significant correlation between IT-enabled dynamic capability and a firm's innovative capability, which in turn, enhances organizational performance.

Except for the aforementioned studies, Ralston et al. [34] and Mentzer et al. [43] have classified logistics service capabilities in terms of demand and supply management interfaces, with the objective of enhancing customer satisfaction. These studies emphasized the importance of a unified logistics theory within the context of the strategic roles and capabilities of the logistics industry. However, Lyu et al. [44] adopted a different approach by developing SEM to explore the impact of diverse logistics resources and patterns, including logistics infrastructure, location, knowledge, and logistics information, on logistics capabilities and operational performance. Jafari et al. [15], investigated the influence of SC flexibility on customer responsiveness and found that SC flexibility positively affects customer responsiveness. The literature review indicates that no efforts have yet been made to identify, analyze, and prioritize key LCFs. Furthermore, none of the aforementioned studies applied ISM and Cross-Impact Matrix Multiplication Applied to Classification (MICMAC) as methodologies to identify, rank, and disclose the interdependence among LCFs. Accordingly, this study aims to address this gap in the field of logistics capability research. Such pioneering research will undoubtedly prove invaluable to the logistics industries and manufacturers.

To this end, an ISM and MICMAC analysis are preferred methodologies for this research. The primary objective of this study is to identify the complex interrelationships among LCFs and construct contextual relationships among them. To achieve this goal, ISM is proposed as the more suitable method. This approach integrates qualitative and interpretive techniques to map the interrelationships among various attributes [22,41] and establish order and direction based on the complexity of the relationships [20,45]. The fundamental concept of ISM is based on the decomposition of complex systems into their constituent subsystems, informed by the practical experience and knowledge of experts [41]. It assists in comprehending the interactions and design patterns of words and graphics among various factors [22,45]. It identifies the influences and elucidates the relationships among the system's attributes. It establishes connections among specific attributes to define a problem based on its dependencies and driving power [20,22], thereby facilitating comprehension of the relative importance of decision variables in uncertain environments [20]. Furthermore, ISM has been widely employed in management, business, economics, and operational research, including studies on SC and logistics, due to its interpretive flexibility [45]. Notable contributions to this field include the works of Ahmadi et al. [46], Bobadilla et al. [47], Bouzon et al. [45], Hashemi Petrudi et al. [5], Singh et al. [48], and Zhong et al. [20]. Interestingly, none of the aforementioned studies employed ISM and MICMAC as a methodology to identify, rank, and elucidate the interdependence among LCFs. Accordingly, this study seeks to address this gap in the existing literature.

### 2.1 Logistics capabilities factors

In the context of the growth of bilateral trade, the increase in demand from e-commerce operators, and the ease of global trade, it is essential that the logistics market has to remain balanced [4]. This has led to a surge of interest in logistics capability as a research topic in the academic field [25]. Logistics capability represents a critical component of a firm's success. It can therefore be defined as a capacity to adopt, integrate, and configure organizational resources in order to respond to challenges. The key capability factors are presented in Table 2, and they are briefly discussed below.

**Table 2:** Logistics capability factors extracted from literature.

Aspects	Capability factors	References
**Service capability**	Demand management interface (DMI)	Evangelista et al. [49]; Gligor and Holcomb [7,37]; Huang and Huang [10]; Jafari et al. [15]; Lyu et al. [44]; Mentzer et al. [43]; Puspitasari and Kusumawardhani [28]; Ralston et al. [34]; Sun et al. [25]; Zhao et al. [30]
	Supply management interface (SMI)	
	Customer-focused based capability (CFBC)	
	Agility & responsiveness (AGR)	
**Innovation capability**	Technological innovation (TI)	Huang and Huang [10]; Jafari et al. [15]; Puspitasari and Kusumawardhani [28]; Ralston et al. [34]; Wang et al. [53], Yang et al. [29]
	Administrative or managerial (AM)	
**Information management capability**	Information technology (IT)	Evangelista et al. [49]; Gligor and Holcomb [37]; Higginson and Alam [39]; Kirono et al. [17]; Lim et al. [41]; Moberg et al. [40]; Rodrigues et al. [54]; Shang and Marlow [18]; Zhao et al. [30]
	Information sharing (IS)	
	Quality information (QI)	
	Commitment to advance information technology (CAIT)	
**Flexibility capability**	Unexpected circumstances (UC)	Evangelista et al. [49]; Huang and Huang [10]; Jafari et al. [15]; Coşkun and Erturgut [56], Moberg et al. [40]; Shang and Marlow [18], Wang et al. [53]; Sinkovics and Roath [19]
	Speculation or anticipation (SA)	
	Operational flexibility (OF)	
**Benchmarking capability**	Internal benchmarking (IBM)	Bagchi [6]; Bagchi [8]; Huang and Huang [10]; Shang [35]; Shang and Marlow [18]
	Competitive benchmarking (CPB)	
	Cooperative benchmarking (COB)	

*Service capability:* Logistics service capabilities are the complex bundle of skills, attributes, abilities, and organizational processes that enable firms to achieve superior performance and competitive advantages over other firms. The development of service capabilities, particularly within the context of the logistics industry, has emerged as a strategic issue [46]. The capabilities derived from the demand management interfaces (DMI) represent a set of best practices for quality logistics, with the objectives of reducing costs [25]. In order to meet the logistics service requirements of their customers, it is incumbent upon logistics operators and service providers to develop their capabilities [51]. These capabilities are associated with customer service, customer satisfaction, time advantages, quick responsiveness to market demand [7,25,37], and product or service differentiation [43], in line with customer standards and the ability to maintain positive customer relationships [44]. In contrast, the capability of the supply management interface (SMI), also known as the supply-oriented or operations-oriented approach, is related to a firm's operational capabilities. It emphasizes product availability, convenience, and minimization of total distribution costs [7,25,37]. The ability to minimize costs is fundamental to the supply management capability of a firm, enabling the identification of proactive, timely and strategic logistics solutions to emergency or customer-specific problems [43]. Thus, this capability constitutes an indispensable foundation for the reduction of costs and capital in context of logistics operations.

*Innovation capability:* To mitigate the impact of SC disruption and effectively manage the associated risk factors, it is essential to leverage innovation as a means of reconfiguring the evolving business environments and navigating the inherent uncertainty associated with logistics [26]. The ability to transform knowledge and ideas into new products, services, processes, and systems for the benefits of a firm is referred to as innovation capability [26,52,53]. In order to gain a competitive advantage and superior performance in a global and dynamic marketplace [29,52], it is essential to enhance the resilience in SC [20]. Consequently, innovation is regarded as a catalyst for navigating volatile business environments [11]. It enables the conduct and coordination of logistics-related activities, as well as the utilization of related resources and skills to fulfil customers' actual needs [11,53]. New technologies, products, and services are typically regarded as technological innovation [27,29], whereas new procedures, policies, and organizational forms are viewed as administrative innovation [27,29]. The concept of innovation in SCM suggests a greater use of external knowledge [38] and the collaborative propensity on the part of a firm to innovate and respond to market changes and customer demand [42]. The integration of innovation capabilities into logistics activities enables LSPs and logistics industries to achieve greater consistency in the operational field logistics [28]. Consequently, the outcomes of technological and managerial innovation in the logistics sector guarantee sustained competitive advantages [34] and mitigate SC risk factors [26].

*Information management capability:* The ever-changing nature of global business makes the ability to manage information more crucial than ever [36]. The advancement of information technology has enabled these elements to achieve enhanced efficiency within the SC [54], while also furnishing insight into evolving and emerging markets [55]. The information management capability of a logistics firm is comprised of four key elements: information technology (IT), information sharing (IS), commitment to advance information technology (CAIT), and information quality (IQ) [7,18,40]. The timely and meaningful sharing of both formal and informal information between firms plays a significant role in the incorporation of logistics capabilities, while communication among firms is necessary for the implementation of knowledge-sharing and long-term and short-term planning [7,39]. The frequent sharing of information among SC members allows quick responses to change and creates better customer-focused capability [39]. Logistics managers, therefore, exchange information between manufacturers and service providers. Commitment to technology such as electronic data interchange (EDI), big data analytics (BDA), and the internet can greatly enhance firms’ ability to increase the speed of information exchange [40], and investment in advanced communication technology can improve the determinants of the quality of information, such as information accuracy and proper formatting.

*Flexibility capability:* In order to respond to or react to uncertainty and change [18,56] and meet customers' demand in market dynamics within low costs, efficient time, and minimal efforts [19,32], it is necessary to implement proactive features, such as the capability of logistics flexibility. This reflects the capacity of service providers to exercise control over circumstances and anticipate potential risks [10,18,53]. It is a fundamental capability of a firm to customize products in accordance with customers' specifications. While firms employ various forms of SC flexibility, including strategic, manufacturing, and marketing flexibility, logistics flexibility, which infers an integrative and customer-oriented perspective, is effective in providing global distribution coverage. In essence, the implementation of logistics flexibility can facilitate enhancements in operational efficiency, speed, volume, and distribution location in accordance with the fluctuations in market demand [32]. The flexibility of purchasing, supply, distribution, and demand management represents a proactive feature in the SC that should possess the requisite degree of flexibility to adapt to changes in ship-to-location [56] and exert control over the degree of uncertainty. Logistics flexibility, therefore, is defined as a sub-criteria of logistic agility [56]. A pull-based logistics system, however, provides a robust foundation for flexibility. Pull processes are characterized by responsiveness to customer demand [10,18], whereas push processes are defined by speculative or anticipation-based processes [57]. Logistics flexibility is effectively represented by a firm's operational flexibility in addressing inventory shortages, responding to customers' short-term fluctuations in demand or supply, and mitigating issues that arise in the production process due to product modification [19].

*Benchmarking capability:* Logistics benchmarking is a process that involves the measurement and comparison of an organization's logistics performance metrics. This enables managers to search for and compare the best practices and/or processes against established standards, industry best practices, and top competitors [6,8,58]. The objective of benchmarking is to gain insight from and integrate processes and innovations that have already been proven effective in other organizations. Consequently, the process of benchmarking offers the potential for the acquisition of knowledge and the implementation of optimal business practices. The benchmarking capability of a firm can be compared with that of internal or external departments to identify areas of strength and weakness. It, therefore, plays a pivotal role in performance evaluation, process reengineering, and the identification of best practices [35,58] and helps mitigate supply chain disruptions by identifying high-value and actionable insights [59]. Logistics benchmarking capability can be classified into three categories: internal benchmarking (IB), competitive benchmarking (CPB), and cooperative benchmarking (COB). In IB, firms relate the performance of internal business units that are engaged in analogous operations, or those that operate in disparate geographical regions [35]. Competitive benchmarking entails a comparison of an organization's performance with industry standards or those of competitors, with the objective of identifying the organization's direct competitors in the marketplace [6,35]. The aim is to emulate and exceed the performance of the “best in class” organizations [8]. In cooperative benchmarking, an organization compares its performance with that of the best-in-class companies in the market [6,35].

## 3 Methodology and data analysis

**Fig. 1:**
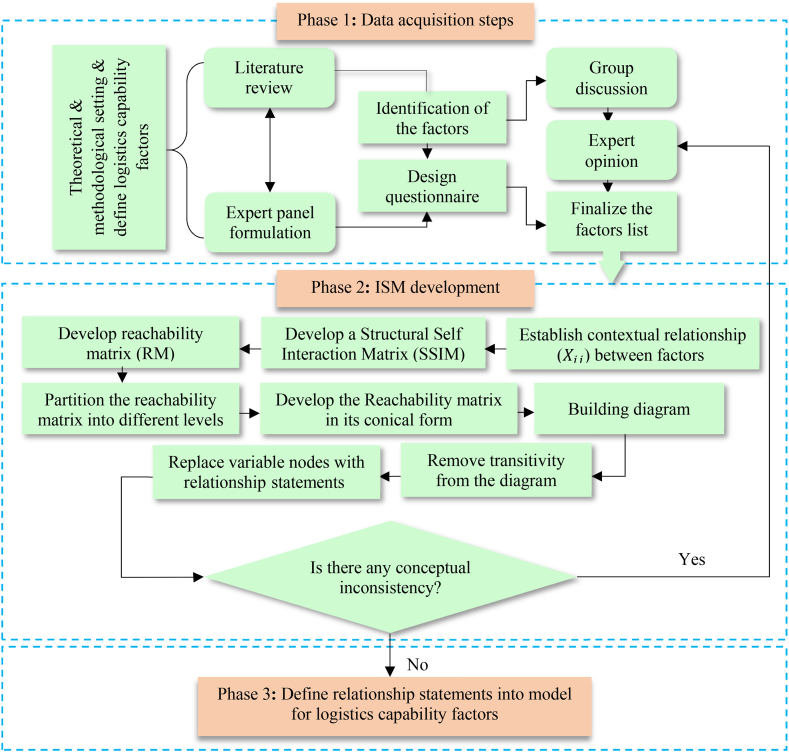
Data acquisition and ISM development procedures.

The methodological part of this research is divided into two sections: data collection and ISM development, along with a MICMAC presentation. Firstly, it forms an expert panel and designs the data extraction procedures. Secondly, the expert's responses are used to build up the structural self-interactive matrix (SSIM) in the ISM approach. Together with ISM, a MICMAC is structured based on the driving and dependency power of the LCFs. The data extraction processes and the methodological framework are shown in Fig. 1.

### 3.1 Data collection procedures

A group of diverse panels of eight experts (group leaders) was formed to conduct this research. The panel of experts was formed in early March 2024. They were formally requested to give their informed consent to participate in this research based on the details of the research topic and purpose. Before the further cooperation, we assured them regarding their personal interests and anonymity. Thus, they participated spontaneously and the eight experts gave their consent for voluntary cooperation. Each of the eight experts is the group leader of their respective team. The eight experts we selected are authorities in eight fields, including university professors in different regions, managers of logistics companies, and research centers. The experts we have selected broadly represent the domain. The experts have a minimum of 10 years of experience in their respective fields. Much more importance has been given to their practical experience in selecting the panel experts. The industry experts are selected based on their practical experience in logistics operations and SCM activities. Although the research has concentrated more on industrial expertise, academic experts are considered as well. The academic experts are selected based on their teaching, research direction, and long-term experience. Previous studies by Ahmadi et al. [46], Hashemi Petrudi et al. [5], and Rezaei et al. [60] have shown that expert-based methodologies can be effective and reach the standard even with a small sample size. To ensure that the results are transparent, reliable, and credible, strict ethical issues and research norms are followed when collecting data. Methodological precision and response bias concerns are the two key challenges that can affect the reliability and credibility of a study's results [61,62]. To overcome the subjectivity and bias issue, expert respondents were granted anonymity at the initial stage of the LCFs selection process, encouraging the experts to provide genuine responses without any fear of being identified [62]. The demographic information of experts is presented in Table 3, while their identities are not disclosed for anonymity and ethical concerns.

**Table 3:** Experts’ profile.

**Experts NO.**	Academic degree	Position	Area of expertise	Experience (Years)	Affiliation
**1**	Master	Operations manager	Operations management	14	Industry
**2**	Master	Transportation manager	Transportation & freight management	13	Industry
**3**	Bachelor	Logistics manager	Distribution management	13	Industry
**4**	Ph D	Associate professor	SC management	11	University
**5**	Ph D	Professor	Transportation & logistics	17	University
**6**	Master	Operations Manager	Distribution & freight management	12	Industry
**7**	Ph D	Operations analyst	Process improvement & quality control	12	Industry
**8**	Ph D	Researcher	Operations-purchasing & procurement	11	Industry

The first step in the data curation process is the identification of LCFs. Prior to extracting experts' final responses, a literature review is applied to the Scopus database using different keywords, literature searching strings, and Boolean operators. The scientific papers that focused on LCFs were finally selected to better define the capability factors. Based on the literature, keyword searches, and experts’ opinions, an initial identification of the sixteen capability factors on the five aspects shown in Table 2 is disclosed to the experts. Then, a group discussion and an initial brainstorming session were conducted to get in-depth insights regarding LCFs. The first phase of the procedures ends in the mid-March 2024 with the selection of 16 factors, primarily. The selection of these capability factors was based on their frequent appearance and occurrence in the literature [20], expert discussion, and their applicability to SCM.

A closed questionnaire is designed following the methodological approach applied by Hashemi Petrudi et al. [5] to refine and customize the primary list of 16 factors in the second step. The questionnaire was extended to each group leader, requesting them to submit their blind response to capability factor selection, identifying which of the factors is more relevant to logistics capability by indicating (✓) as accept and ( × ) as reject. The responses of eight experts are the conclusions of their own teams after careful consideration, not just their own opinions. Therefore, the responses we use are not just the ideas of eight experts but the ideas of their teams from various areas of logistics expertise to represent other experts in the field of logistics as much as possible. The authors had another session with the experts and decided that the only factors approved by at least six decision-makers would be selected for the final list. The same procedures have been applied in some prior studies [5,46]. The experts' responses in this phase were completely anonymized and blind to minimize the possibility of bias. The expert's opinion is presented in Table 4.

**Table 4:** Deciding logistics capability factors (LCFs) based on experts’ response.

SL.	Capability factors	E1	E2	E3	E4	E5	E6	E7	E8	Decision
**1**	Demand management interface	✓	✓	✓	✓	✓	✓	✓	✓	Selected
**2**	Supply management interface	✓	✓	✓	✓	✓	✓	✓	✓	Selected
**3**	Customer-focused based capability	✓	×	✓	×	✓	✓	×	×	Rejected
**4**	Agility & responsiveness	✓	✓	✓	✓	✓	×	✓	✓	Selected
**5**	Technological innovation	✓	✓	✓	✓	✓	✓	✓	✓	Selected
**6**	Administrative or managerial	✓	×	✓	×	×	×	✓	✓	Rejected
**7**	Information technology	✓	✓	✓	✓	✓	✓	×	✓	Selected
**8**	Information sharing	✓	✓	✓	✓	✓	✓	✓	×	Selected
**9**	Quality information	✓	✓	✓	✓	✓	✓	✓	×	Selected
**10**	Commitment to advance information technology	✓	✓	✓	✓	✓	✓	✓	✓	Selected
**11**	Unexpected circumstances	✓	✓	✓	✓	×	✓	✓	✓	Selected
**12**	Speculation or anticipation	×	×	×	✓	✓	×	✓	✓	Rejected
**13**	Operational flexibility	✓	✓	✓	✓	✓	✓	✓	✓	Selected
**14**	Internal benchmarking	✓	✓	×	✓	✓	✓	✓	✓	Selected
**15**	Competitive benchmarking	×	✓	✓	✓	✓	✓	✓	✓	Selected
**16**	Cooperative benchmarking	✓	×	×	✓	✓	×	✓	✓	Rejected

[**Note-** E1, E2, …E8; Expert one - eight].

Four factors are omitted out of sixteen. The resulting twelve capability factors are finalized based on the expert's response presented in Table 5. Finally, the experts are invited once again to participate in the final session of an open group discussion at the end of March 2024. The primary aim of this session is to define the interrelationships among the LCFs listed in Table 5. In addition, this open discussion will allow an in-depth exploration of the topic [5]. The session was formally started by introducing the experts. Then, the research objective, scope, and capability factors were briefly explained. Finally, the experts were requested to give their opinions about the interrelationships among the LCFs as a part of developing the structural self-interactive matrix (SSIM) in the ISM-MICMAC approach, which is the methodology of building interdependencies among the factors.

**Table 5:** List of the finalized factors.

SL.	Capability factors	Code
**1**	Demand management interface	DMI
**2**	Supply management interface	SMI
**3**	Agility & responsiveness	AGR
**4**	Technological innovation	TI
**5**	Information technology	IT
**6**	Information sharing	IS
**7**	Quality information	QI
**8**	Commitment to advance information technology	CAIT
**9**	Unexpected circumstances	UC
**10**	Operational flexibility	OF
**11**	Internal benchmarking	IBM
**12**	Competitive benchmarking	CPB

### 3.2 ISM-MICMAC development and results analysis

There are various alternative methods to study the interrelationship among the attributes. Analytical Hierarchy Process (AHP), Analytical Network Process (ANP), Decision Making Trial and Evaluation Laboratory (DEMATEL), Best Worst Method (BWM), and Structural Equation Modeling (SEM) [5] are notable in this regard. AHP, ANP, and BWM require pairwise comparisons [5], while DEMATEL establishes cause-and-effect interactions among the factors [20]. SEM, a multivariate technique, only measures the direct and indirect effects of putative causal relationships [63]. Only interpretive structural modeling (ISM) particularly focuses on experts’ experience and knowledge in defining the contextual relationships of a complicated system [45]. The rationale for using the ISM-MICMAC approach lies in its ability to handle complex and multiple relationships among variables that exist in this research [20]. It allows not only to define the capability factors but also prioritize those factors based on their driving and dependency power. Additionally, it demonstrates a structure of the relationships between these factors, supporting the understanding and interpretation of our results. These led to the choice of ISM as methodology.

Regarding the validation and validity analysis of our model, we followed Hashemi Petrudi et al. [5] to conduct a two-step process. First, we performed an internal validity check to ensure the accuracy and consistency of our results [5]. We cross-validated our results with the input data and ran several iterations until we reached a stable model. Secondly, we compared our research findings with existing literature and empirical evidence for external validity [5]. A high degree of alignment between our findings and previous research is found, which strengthens the validity of the results.

After modeling the ISM, a MICMAC is structured based on the driving and dependency power of the LCFs. The different phases of ISM are as follows-

***Phase 1 Identify capability factors*:** identify and determine LCFs through a literature review, group discussion, and brainstorming. This research finalized twelve capability factors based on five aspects of logistics performance, as shown in Table 2.

***Phase 2 Develop contextual relationships and build SSIM*:** develop the contextual relationships among capability factors and build up a structural self-interaction matrix (SSIM) [45,46]. Four symbols are used to develop the SSIM:

V: factor $X_{i}$ will influence the factor $X_{j}$.

A: factor $X_{j}$ will influence the factor $X_{i}$.

X: factor $X_{i}$ and factor $X_{j}$ will influence each other.

O: factor $X_{i}$ and factor $X_{j}$ are unrelated.

Based on the contextual relationships among the factors, the SSIM has been developed, as shown in Table 6.

**Table 6:** Structural self-interactive matrix (SSIM).

SL.	Capability factors	Code	12	11	10	9	8	7	6	5	4	3	2
**1**	Demand management interface	DMI	O	O	V	O	O	O	V	O	O	O	V
**2**	Supply management interface	SMI	O	O	V	A	A	O	O	O	O	A	
**3**	Agility & responsiveness	AGR	O	O	O	O	O	O	A	A	O		
**4**	Technological innovation	TI	V	V	O	O	V	V	O	X			
**5**	Information technology	IT	O	O	V	O	V	X	X				
**6**	Information sharing	IS	O	O	O	O	A	A					
**7**	Quality information	QI	O	O	V	O	X						
**8**	Commitment to advance information technology	CAIT	O	O	V	O							
**9**	Unexpected circumstances	UC	O	O	X								
**10**	Operational flexibility	OF	V	O									
**11**	Internal benchmarking	IBM	X										
**12**	Competitive benchmarking	CPB											

***Phase 3 Develop reachability matrix*:** the SSIM needs to be converted into a binary matrix called the initial reachability matrix [45]. The reachability matrix is the path that indicates whether one capability factor can establish a path of arrival with another factor through the indirect action of other factors [21,45,46,48]. By replacing V, A, X, and O with 1 and 0, the following rules are applied to get the initial reachability matrix [21,45].

■ If $X_{ij}$ entry in SSMI is V, then $X_{ij}$ entry in the reachability matrix becomes 1 and the $X_{ji}$ entry becomes 0.
■ If $X_{ij}$ entry in SSMI is A, then $X_{ij}$ entry in the reachability matrix becomes 0 and the $X_{ji}$ entry becomes 1.
■ If $X_{ij}$ entry in SSMI is X, then $X_{ij}$ entry in the reachability matrix becomes 1 and the $X_{ji}$ entry also becomes 1.
■ If $X_{ij}$ entry in SSMI is O, then $X_{ij}$ entry in the reachability matrix becomes 0 and the $X_{ji}$ entry also becomes 0.

The initial reachability matrix is shown in Table 7. The final reachability matrix (FRM), shown in Table 8, is constructed from the initial reachability matrix by incorporating the transitivity rules, where transitivity is marked as 1∗ [21]. Transitivity is a relationship between three elements; for example, if there is a relationship between factor A and factor B, and B and C, then there is automatically a transitive relationship between factor A and C [21].

**Table 7:** Initial reachability matrix (IRM).

Capability factors	Code	1	2	3	4	5	6	7	8	9	10	11	12
Demand management interface	DMI	1	1	0	0	0	1	0	0	0	1	0	0
Supply management interface	SMI	0	1	0	0	0	0	0	0	0	1	0	0
Agility & responsiveness	AGR	0	1	1	0	0	0	0	0	0	0	0	0
Technological innovation	TI	0	0	0	1	1	0	1	1	0	0	1	1
Information technology	IT	0	0	1	1	1	1	1	1	0	1	0	0
Information sharing	IS	0	0	1	0	1	1	0	0	0	0	0	0
Quality information	QI	0	0	0	0	1	1	1	1	0	1	0	0
Commitment to advance information technology	CAIT	0	1	0	0	0	1	1	1	0	1	0	0
Unexpected circumstances	UC	0	1	0	0	0	0	0	0	1	1	0	0
Operational flexibility	OF	0	0	0	0	0	0	0	0	1	1	0	1
Internal benchmarking	IBM	0	0	0	0	0	0	0	0	0	0	1	1
Competitive benchmarking	CPB	0	0	0	0	0	0	0	0	0	0	1	1

**Table 8:** Final reachability matrix (FRM).

Capability factors	Code	1	2	3	4	5	6	7	8	9	10	11	12	Driving power
Demand management interface	DMI	1	1	1∗	1∗	1∗	1	1∗	1∗	1∗	1	1∗	1∗	12
Supply management interface	SMI	0	1	0	0	0	0	0	0	1∗	1	1∗	1∗	5
Agility & responsiveness	AGR	0	1	1	0	0	0	0	0	1∗	1∗	1∗	1∗	6
Technological innovation	TI	0	1∗	1∗	1	1	1∗	1	1	1∗	1∗	1	1	11
Information technology	IT	0	1∗	1	1	1	1	1	1	1∗	1	1∗	1∗	11
Information sharing	IS	0	1∗	1	1∗	1	1	1∗	1∗	1∗	1∗	1∗	1∗	11
Quality information	QI	0	1∗	1∗	1∗	1	1	1	1	1∗	1	1∗	1∗	11
Commitment to advance information technology	CAIT	0	1	1∗	1∗	1∗	1	1	1	1∗	1	1∗	1∗	11
Unexpected circumstances	UC	0	1	0	0	0	0	0	0	1	1	1∗	1∗	5
Operational flexibility	OF	0	1∗	0	0	0	0	0	0	1	1	1∗	1	5
Internal benchmarking	IBM	0	0	0	0	0	0	0	0	0	0	1	1	2
Competitive benchmarking	CPB	0	0	0	0	0	0	0	0	0	0	1	1	2
Dependence power		1	10	7	6	6	6	6	6	10	10	12	12	

Later, the reachability set and the antecedent set are derived from the final reachability matrix [41]. The reachability set for an individual's factor consists of itself and the other factor that it also tries to achieve, while its antecedent set consists of the factor itself and the other factor that may also try to achieve it [22,41,45,64]. Based on Lim et al. [41]*, ReM*^*X*^ = *[rm*_*ij*_*]*_*n×n*_ indicates the nth expert's individual reachability matrix. Then the total reachability matrix *R*^*T*^ can be calculated:

(1) $$R^{T}=\frac{1}{n}\left({rm}_{ij}^{1}+{rm}_{ij}^{2}+\ldots +{rm}_{ij}^{n}\right),i,j=1,2,3,\ldots n$$

The reachability $\left(R\overset{´}{e}\right)$ and the antecedent $\left(A\overset{´}{t}\right)$ set can be determined from the total reachability matrix as:

(2) $$r_{i}=1,R\overset{´}{e}=\left\{{rm}_{1}^{R\overset{´}{e}},{rm}_{2}^{R\overset{´}{e}},\ldots {rm}_{n}^{R\overset{´}{e}}\right\}$$

(3) $$r_{j}=1,A\overset{´}{t}=\left\{{rm}_{1}^{A\overset{´}{e}},{rm}_{2}^{A\overset{´}{e}},\ldots {rm}_{n}^{A\overset{´}{e}}\right\}$$

An intersection set between reachability and the antecedent can be generated as follows:

(4) $$Í=\overset{´}{R}\cap \overset{´}{A}$$

***Phase 4 Level partitions:*** the reachability and the antecedent set are used in the level partitioning [22,64]. The reachability set consists of the horizontal factors, while the antecedent set consists of the vertical factors [21]. The intersection between these two sets is then derived for all factors. The factors for which the reachability and the intersection sets are the same are placed at the top level of the ISM hierarchy [21,22,45,64]. The top-level factors placed in the hierarchy would not help achieve any other factors above their level. The top-level factors are then identified and separated from the other factors for the next level of iteration. The same process is repeated until the level of each factor is determined [16,22,41]. Thus, this helps to construct the diagram. The level partitioning of the capability factors with the reachability set, antecedent set, and intersection set is shown in Table 9.

**Table 9:** Level partition (Iteration 1–5).

Factor	Reachability set (R)	Antecedent set (A)	Intersection set (R∩A)	Level
**1**	1, 2, 3, 4, 5, 6, 7, 8, 9, 10, 11, 12	1	1	
**2**	2, 9, 10, 11, 12	1, 2, 3, 4, 5, 6, 7, 8, 9, 10	2, 9, 10	
**3**	2, 3, 9, 10, 11, 12	1, 3, 4, 5, 6, 7, 8	3	
**4**	2, 3, 4, 5, 6, 7, 8, 9, 10, 11, 12	1, 4, 5, 6, 7, 8	4, 5, 6, 7, 8	
**5**	2, 3, 4, 5, 6, 7, 8, 9, 10, 11, 12	1, 4, 5, 6, 7, 8	4, 5, 6, 7, 8	
**6**	2, 3, 4, 5, 6, 7, 8, 9, 10, 11, 12	1, 4, 5, 6, 7, 8	4, 5, 6, 7, 8	
**7**	2, 3, 4, 5, 6, 7, 8, 9, 10, 11, 12	1, 4, 5, 6, 7, 8	4, 5, 6, 7, 8	
**8**	2, 3, 4, 5, 6, 7, 8, 9, 10, 11, 12	1, 4, 5, 6, 7, 8	4, 5, 6, 7, 8	
**9**	2, 9, 10, 11, 12	1, 2, 3, 4, 5, 6, 7, 8, 9, 10	2, 9, 10	
**10**	2, 9, 10, 11, 12	1, 2, 3, 4, 5, 6, 7, 8, 9, 10	2, 9, 10	
**11**	11, 12	1, 2, 3, 4, 5, 6, 7, 8, 9, 10, 11, 12	11, 12	1
**12**	11, 12	1, 2, 3, 4, 5, 6, 7, 8, 9, 10, 11, 12	11, 12	1
**Iteration 2**				
**1**	1, 2, 3, 4, 5, 6, 7, 8, 9, 10	1	1	
**2**	2, 9, 10	1, 2, 3, 4, 5, 6, 7, 8, 9, 10	2, 9, 10	2
**3**	2, 3, 9, 10,	1, 3, 4, 5, 6, 7, 8	3	
**4**	2, 3, 4, 5, 6, 7, 8, 9, 10	1, 4, 5, 6, 7, 8	4, 5, 6, 7, 8	
**5**	2, 3, 4, 5, 6, 7, 8, 9, 10	1, 4, 5, 6, 7, 8	4, 5, 6, 7, 8	
**6**	2, 3, 4, 5, 6, 7, 8, 9, 10	1, 4, 5, 6, 7, 8	4, 5, 6, 7, 8	
**7**	2, 3, 4, 5, 6, 7, 8, 9, 10	1, 4, 5, 6, 7, 8	4, 5, 6, 7, 8	
**8**	2, 3, 4, 5, 6, 7, 8, 9, 10	1, 4, 5, 6, 7, 8	4, 5, 6, 7, 8	
**9**	2, 9, 10	1, 2, 3, 4, 5, 6, 7, 8, 9, 10	2, 9, 10	2
**10**	2, 9, 10	1, 2, 3, 4, 5, 6, 7, 8, 9, 10	2, 9, 10	2
**Iteration 3**				
**1**	1, 3, 4, 5, 6, 7, 8	1	1	
**3**	3	1, 3, 4, 5, 6, 7, 8	3,	3
**4**	3, 4, 5, 6, 7, 8	1, 4, 5, 6, 7, 8	4, 5, 6, 7, 8	
**5**	3, 4, 5, 6, 7, 8	1, 4, 5, 6, 7, 8	4, 5, 6, 7, 8	
**6**	3, 4, 5, 6, 7, 8	1, 4, 5, 6, 7, 8	4, 5, 6, 7, 8	
**7**	3, 4, 5, 6, 7, 8	1, 4, 5, 6, 7, 8	4, 5, 6, 7, 8	
**8**	3, 4, 5, 6, 7, 8	1, 4, 5, 6, 7, 8	4, 5, 6, 7, 8	
**Iteration 4**				
**1**	1, 4, 5, 6, 7, 8	1	1	
**4**	4, 5, 6, 7, 8	1, 4, 5, 6, 7, 8	4, 5, 6, 7, 8	4
**5**	4, 5, 6, 7, 8	1, 4, 5, 6, 7, 8	4, 5, 6, 7, 8	4
**6**	4, 5, 6, 7, 8	1, 4, 5, 6, 7, 8	4, 5, 6, 7, 8	4
**7**	4, 5, 6, 7, 8	1, 4, 5, 6, 7, 8	4, 5, 6, 7, 8	4
**8**	4, 5, 6, 7, 8	1, 4, 5, 6, 7, 8	4, 5, 6, 7, 8	4
**Iteration 5**				
**1**	1	1	1	5

In Table 9, capability factors 11 (internal benchmarking) and 12 (competitive benchmarking) are found at level 1. This places them at the top of the hierarchy. After removing factors 11 and 12, the second iteration continues, and factor 2 (supply management interface), 9 (unexpected circumstances), and 10 (operational flexibility) are at level 2. This places them on the second level of the hierarchy. Similarly, factor 3 (agility and responsiveness) is levelled at 3 and positioned at 3; factor 4 (technological innovation), 5 (information technology), 6 (information sharing), 7 (quality information), and 8 (commitment to advance information technology) are levelled at 4 and positioned at the fourth level of the hierarchy; and finally, factor 1 (demand management interface) is levelled at 5 and positioned at the bottom of the hierarchy of the ISM model.

**Fig. 2:**
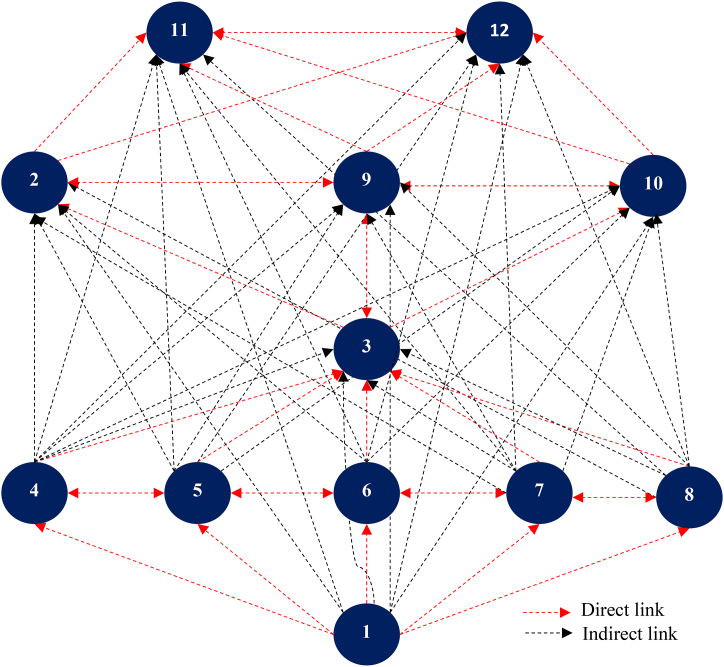
Digraph of logistics capability factors.

***Phase 5 Development of conical matrix and digraph:*** the conical matrix is developed from the partitioned reachability matrix by rearranging the LCFs according to their level, i.e., all factors with the same level are pooled together. The driving and dependency power of the capability factors are also calculated. The conical matrix is shown in Table 10, which eventually becomes a structure called a digraph. In the digraph development process, the top-level capability factors are positioned at the top of the digraph, the second-level capability factors are positioned at the second place, and so on, until the bottom level is placed at the lowest position in the digraph, as shown in Fig. 2.

**Table 10:** Conical matrix (CM).

Capability factors	11	12	2	9	10	3	4	5	6	7	8	1	**Driving power**	**Level**
11	1	1	0	0	0	0	0	0	0	0	0	0	2	1
12	1	1	0	0	0	0	0	0	0	0	0	0	2	1
2	1∗	1∗	1	1∗	1	0	0	0	0	0	0	0	5	2
9	1∗	1∗	1	1	1	0	0	0	0	0	0	0	5	2
10	1∗	1	1∗	1	1	0	0	0	0	0	0	0	5	2
3	1∗	1∗	1	1∗	1∗	1	0	0	0	0	0	0	6	3
4	1	1	1∗	1∗	1∗	1∗	1	1	1∗	1	1	0	11	4
5	1∗	1∗	1∗	1∗	1	1	1	1	1	1	1	0	11	4
6	1∗	1∗	1∗	1∗	1∗	1	1∗	1	1	1∗	1∗	0	11	4
7	1∗	1∗	1∗	1∗	1	1∗	1∗	1	1	1	1	0	11	4
8	1∗	1∗	1	1∗	1	1∗	1∗	1∗	1	1	1	0	11	4
1	1∗	1∗	1	1∗	1	1∗	1∗	1∗	1	1∗	1∗	1	12	5
**Dependence power**	12	12	10	10	10	7	6	6	6	6	6	1		
**Level**	1	1	2	2	2	3	4	4	4	4	4	5

**Fig. 3:**
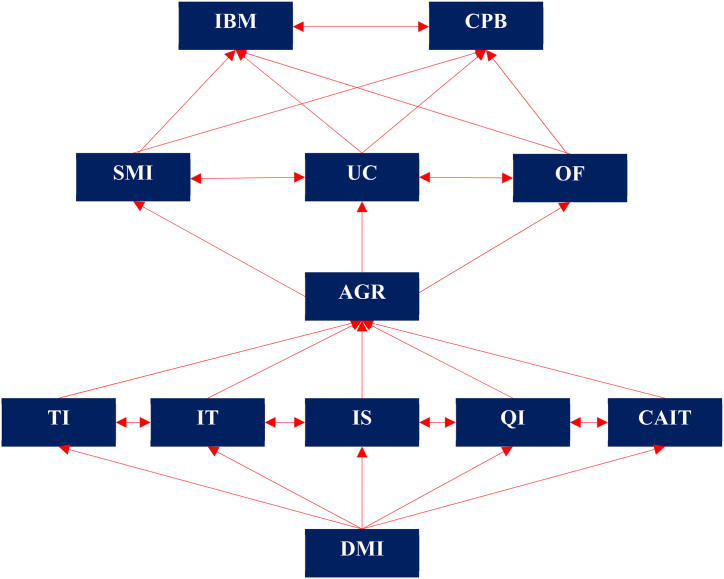
ISM of logistics capability factors.

***Phase 6 Development of ISM model*:** the initial digraph (Fig. 2) includes transitivity links (indirect links). The digraph is finally transformed into the ISM model after removing the transitivity links. The nodes of the digraph are also replaced by the capability factors to highlight the relationship statements, as shown in Fig. 3. It is significant to see that the demand management interface (DMI) of service capability and information management capabilities play a crucial driving role in improving logistics performance, and they are positioned at levels 5 and 4 of the ISM hierarchy, respectively. Interestingly, logistics capability factors 11 (internal benchmarking, IBC) and 12 (competitive benchmarking, CPB) are the factors that depend on other capability factors for both internal and external improvement. This is why these factors appear at the top of the hierarchy.

The ISM model in Fig. 3 reveals that the DMI of logistics service capability is a high driving power factor that can influence others to improve performance. In fact, the customer's demand-oriented capability emphasizes the external dimensions of the customer, customer interfaces, and the goals and objectives of logistics service coordination. For example, the DMI is related to customer service and responsiveness and incorporates the elements of customer focus, time management, integration, information exchange, and service evaluation. It balances customer requirements with the capabilities of the SMI. As such, DMI is the foundation of the logistics capability structure that initiates all other logistics activities and passes through information and innovation capability for supply management efficiency.

**Fig. 4:**
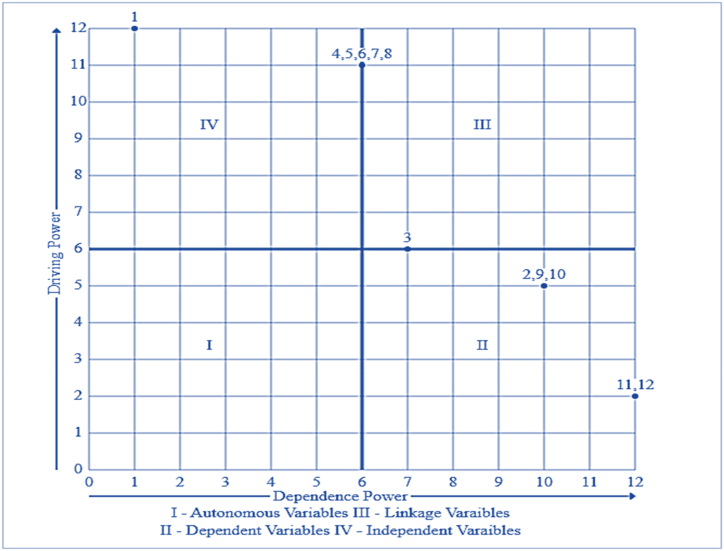
MICMAC analysis of logistics capability factors.

In addition, the MICMAC analysis in Fig. 4 validates the ISM model and categorizes the logistics capability factors. The factors are classified into four clusters based on the driving and dependence powers derived from the Final Reachability Matrix (FRM). To draw the diagram, the driving power of the capability factors is represented as a vertical axis, and the dependence power is represented as the horizontal axis. The capability factors are categorized as autonomous, dependent/dominated, linkage/relay, and independent/dominant.

Cluster 1: Autonomous capability factors: They include variables that have weak driving power and weak dependency [45]. These variables are relatively detached from the system with which they have only a few links, which may be strong. Interestingly, there are no autonomous capability factors disconnected from the complex capability system.

Cluster 2: Dependent capability factors: These consist of variables that have weak driving power but a strong dependency [22,41,45]. Logistics capability factors, such as internal benchmarking (IBM), competitive benchmarking (CPB), supply management interface (SMI), unexpected circumstances (UC), and operational flexibility (OF), are in this cluster.

Cluster 3: Linkage capability factors: They have a strong driving power and a strong dependence. These variables belong to the category of independent or linkage variables [22,41,64]. Any action on these variables will affect others and also have a feedback effect on themselves [62]. Capability factors 3 (Agility and responsiveness, AGR), 7 (Quality information, QI), and 8 (Commitment to advanced information technology, CAIT) are included in this group. Though factors 6 (Information sharing, IS), 7 (Quality information, QI), and 8 (Commitment to advanced information technology, CAIT) occupy an intermediary position and fulfill a mediatory and correlational role in the system, they can impact the factors above them while also being influenced by the ones below them.

Cluster 4: Independent capability factors: these are independent variables with strong driving power but weak dependency [21,45,48]. Capability factors of Demand Management Interface (DMI), Technological Innovation (TI), Information Technology (IT), and Information Sharing (IS) are significant dominating factors that dominate all other elements of firms’ logistics efficiency. Surprisingly, QI, CAIT, IT, IS, and TI can play a dual role both as dominant and linking capability factors. From Fig. 3, decision makers can perceive the complex interrelationships among capability factors and further gain valuable insights for decision-making.

## 4 Discussion and implications

### 4.1 Discussion

The study has demonstrated the intricate interrelationships among LCFs through the integration of ISM and MICMAC analysis. The objectives of the proposed framework were to identify LCFs and to elucidate the interrelationships and interdependencies among them based on their driving and dependence power. The LCFs were defined through a comprehensive literature review and validated by experts. The prioritization of capability factors to enhance logistics performance is critically assessed in the study. The expert knowledge-based integrated approach categorized the LCFs into five hierarchical levels within the ISM digraph, while MICMAC classified them into four distinct categories. The hierarchical digraph of ISM and the MICMAC map illustrate the significance of the LCFs, positioning them at various levels based on their relative power. Thus, the structural framework of this research prioritized LCFs and formulated relevant management implications for SC managers, thereby enriching the literature on capabilities and defining the complex hierarchical relationships among them.

The results of the study identified significant hierarchical relationships among the factors. Logistics performance based on the demand management interface (DMI) initiates the complex interdependency and structures of the proposed capability factor framework. This is followed by technological innovation (TI) and information management capability (i.e., IT, IS, QI, CAIT). These factors have significant driving power in the model and play a crucial role in stimulating firms’ logistics capabilities. The capability factors at lower levels of the ISM model (i.e., DMI, TI, IS, etc.) are the most critical and influential factors. They have more influence on the integrated system and can induce more LCFs. The factors at higher levels (i.e., BMC, CPB) of the hierarchy have less influence on the system and can induce fewer logistics capability factors. These factors with high dependency and low driving power require more attention from logistics managers. Accordingly, the results of our study are consistent with the findings of previous studies by Cheng et al. [65], Dang et al. [66], Huang and Huang [10], Jafari et al. [15], and Nan et al. [67].

The results show that demand management interface (DMI) is a central capability factor in the model and it can significantly drive other capability factors in firms. This is in line with Gligor and Holcomb [7], who emphasized the significance of demand- or customer-oriented services and categorized service management as pre-sales customer service, post-sales customer service, delivery speed, and responsiveness. Therefore, it is seen as the external dimension of the customer and customer interfaces, which is associated with services and responsiveness to market demand [37]. In this case, logistics companies must not only promote superior firm-level logistics capabilities, but they must also be able to integrate their logistics capabilities with those of their service efficiency (demand and supply management) [7]. Furthermore, the findings are consistent with the theory of Mentzer et al. [43], where they observed DMI covering multiple areas of SCM, including inventory, planning, promotion, and customer service. It drives demand and supply and helps smooth the volatility created by higher consumer expectations and shorter SC fulfillment cycles. Customer-centric demand management goes on to include improved service, more accurate forecasting, improvement of existing products, and excellence in new product introductions. Accordingly, the findings of this research place much more emphasis on logistics service differentiation (demand and supply), which is also proved by the study of Ralston et al. [34].

Obviously, the results shown in Fig. 3 are consistent with the previous studies by Puspitasari and Kusumawardhani [28], Cheng et al. [65], and Nan et al. [67]. Our results showed technological innovation (TI) and information management capability (i.e., IT, IS, QI, CAIT) are the dominant capability factors. Practicing these capabilities doesn't only create value for customers but also enhance organizational capability by anticipating and planning demand and supply management and eliminating waste, thus increasing value in every area of logistics service [46]. In their study, Bagheri et al. [23], Cheng et al. [65], and Rajesh [16] proved that technological innovation positively improves logistics development, while the findings of Nan et al. [67] showed a robust positive correlation of logistics innovation with geography to increase the capacity of the logistics industry. All this evidence is consistent with our findings that technological innovation capability is one of the dominant characteristics that is recognized as the core capability of organizations to master and sustain holistic value-creation dynamics [10]. Thus, logistics innovations or analytical capabilities to support customer-centric demand management are what it takes to win in a fast-paced and highly competitive global marketplace. The success of DMI in firms can be achieved by practicing technological innovation (TI) and information management capability. That's why Puspitasari and Kusumawardhani [28] considered TI for logistics capability as a crucial catalyst for a firm's performance and survival. Moberg et al. [40] and Zhao et al. [30] resolved information technology and information quality as two perceived management components for service improvement and as key predictors of firm performance.

The diversification of logistics activities has made logistics firms agile and responsive to new products, reducing production time and the product development cycle. In addition to TI and information management capabilities, the ability of industries to cope with unexpected circumstances (UC) needs to be improved through agility and responsiveness (AGR). Agility enables an increase in the level of responsiveness in firms. Thus, the results of this study show that agility and responsiveness (AGR) are the connecting factors between demand and supply management interfaces. However, logistics firms can face UC arising from customer demand and supply, product modulation, and customization. To meet the unexpected situation, operational flexibility (OF): strategic, manufacturing, and marketing are inevitable [10]. Flexibility in the SCM, a proactive feature, provides more agility in operations in the context of dynamic capability [56]. Therefore, Coşkun and Erturgut [56], in line with our findings, opined that AGR, together with OF, have the capability of operating uncertainty. Fig. 3 shows UC between demand and supply management interfaces depends much more on OF and AGR. These findings are also confirmed by the study of Piprani et al. [32], where the authors pointed out that multidimensional flexibility significantly contributes to improving SC resilience.

The structural model and the MICMAC present that AGR, OF, and UC act as the bridge between demand and supply management interfaces to achieve logistics benchmarks in companies that could help them stand alone in the market. Our results shown in Fig. 4 categorize benchmarking capability as the highly dependent factor. This is because a company's benchmarking success depends on a number of significant factors, including training, internal processes, improved customer service, and improved goal setting [6,18]. Bagchi [8] also confirms that benchmarking involves continuous monitoring and measurement of a company's performance against the “best-in-class” companies. Therefore, logistics managers need to be aware of the potential of internal and competitive benchmarking. This system of LCFs allows companies to pay close attention to pervasive benchmarks, bring them to market, and meet the growing demands of customers through innovative practices, adopting technology, and creating operational, internal, and competitive benchmarks between firms.

The results of this study are unique and valuable because the same approach has not yet been used in LCFs studies. Our results from ISM-MICMAC are quite different from other alternative approaches such as SEM, AHP, DEMTAEL, or entropy weight TOPSIS. The studies of Chen et al. [24], Cheng et al. [31], Fanghu et al. [50], Jafari et al. [15], and Zhong et al. [20] are examples. Thus, the complex model of LCFs provides clear ways to promote logistics performance by bolstering service efficiency based on TI and information management capability. This research therefore makes a significant contribution to current knowledge of LCFs and their interdependency.

### 4.2 Theoretical implications

The findings of this study offer a number of theoretical implications for the interpretation of the relationships between LCFs. Firstly, the synthesis of the literature on LCFs in the SC domain extends the existing body of knowledge through the application of the ISM technique and MICMAC analysis. The combination of ISM and MICMAC allows organizations to gain a comprehensive understanding of their LCFs, thereby enabling them to prioritize their efforts to improve their logistics performance. This structural modeling approach assists in the identification of the principal drivers and dependencies within the logistics system, thereby allowing organizations to concentrate their efforts on the enhancement of the critical factors that have the most significant impact on their overall performance. Consequently, this study is a valuable contribution to the existing body of research exploring the relationship between LCFs and the improvement of logistics performance and provides further support for the findings of earlier studies that have identified a range of effects of capability factors on logistics performance.

Secondly, the ISM-MICMAC technique is widely utilized across various fields for conceptualization and to address essential questions related to theory building. These questions pertain to the nature of the phenomenon in question (i.e., 'what'), the mechanisms by which it operates ('how'), and the underlying reasons for its existence ('why'). In this study, the 'what' question has been answered by identifying LCFs based on a comprehensive review of the literature and expert opinions. The 'how' and 'why' questions have been explored by analyzing the strength and significance of the capability factors through interpretive analysis.

Third, based on the expert's knowledge, the study has identified the most significant capability factors and their interdependencies, which have been central to logistics performance in firms in recent years. The findings revealed the structural interrelationships and dependencies among the logistics capability factors, based on their driving and dependence powers. The proposed framework, utilizing ISM-MICMAC, unveiled a multifaceted hierarchical model for decision-making in prioritizing capability factors. Operations managers can apply the ISM approach and the results as a reference for developing a hierarchy of capability factors at various levels.

Finally, the study reveals that logistics performance is a combination of the service capabilities of DMI and SMI, which can be operationalized through technological innovation (TI), quality information (QI), agility and responsiveness (AGR), and flexible practices. This finding provides valuable insights into logistics capability research and can inform future theoretical and methodological developments.

### 4.3 Managerial implications

This study not only enriches the existing literature, but its implications are of great importance to logistics and operations managers. After the post-pandemic era, this study provides benchmarks to assist SC managers in deciding the LCFs based on their driving and dependency power. Thus, the LCFs' model has a clear significance for policymakers in sorting the capability factors and defining the performance level of their companies based on the hierarchical structure of the ISM. The results of this research don't only assist to sort the LCFs and define their power based on the ISM-MICMAC; it also works as an experience-based reference model for the operations managers to implement in their operations. In addition, the results enable managers to be more cautious about the factors with high dependency and low driving power in their company.

This study contributes to the ongoing complexity of the role of LCFs in improving logistics performance in organizations. Although many scholars have evaluated LCFs in different ways, this study stands alone among them in its unique contribution in building a capability model based on ISM-MICMAC to visualize the hierarchical difference of LCFs, which has great importance for managers in making decisions regarding the performance of their company.

Most importantly, the study allows the practitioners to gain a clear understanding of the complexity inherent in capability factors. The findings focus on improving the service capabilities of the demand and supply management interface through information and innovation capabilities. Thus, operations managers should recognize the critical role of information integration in facilitating seamless information sharing and coordination among partners. By coordinating and sharing information, they enable real-time and accurate information flow, thereby increasing service visibility and transparency. This is because they are charged with setting the right direction for their business. Their digital engagement with information, improved technologies, quality information, and innovation enhance the effectiveness of SCM practices among partners. It is therefore essential for them to pay attention to the diversification of demand management interface (DMI) which our findings showed as crucial for achieving operational flexibility and establishing internal and competitive benchmarks.

In addition, the results of this study help practitioners closely monitor the pitfalls and interdependencies of their organization's LCFs as they implement strategies. As logistics managers today strive to understand the priorities of their own capabilities, they need empirical evidence to enable them to leverage a limited number of essential capability factors to improve their performance. This study provides them with a comprehensive view.

In this case, the ISM-MICMAC model highlights the hierarchical interdependencies among capability factors, which helps logistics operators adopt a practical approach to prioritize low-driving factors that enhance SC competitiveness. Clearly, high dependency and low driving LCFs, such as internal benchmarking (IBM) and competitive benchmarking (CPB), require deep attention from managers in improving logistics performance.

## 5 Conclusion and future research

In the post-pandemic era, SC disruptions and increasing global trade have made logistics activities very complex. The improvement of logistics capabilities through logistics capability factors (LCFs) is of great importance. Therefore, this study aimed to define the significant LCFs and explore their interdependencies and interrelationships, thereby helping SC managers to understand the complex pattern of LCFs. The study used an ISM-MICMAC approach in modeling LCFs based on the experience of experts in exploring the capability factors.

In relation to our research objective, the logistics capability factors are identified and validated based on experts’ experience. The factors are ranked based on their driving and dependency power building a hierarchical ISM. By constructing an ISM-MICMAC diagram, this study examines the relationships and interactions among various logistics capability factors, thereby presenting an evaluation framework that explains the complex interrelationships and interdependencies among LCFs. The results emphasize the importance of the demand management interface, which encompasses service capability, technological innovation, and information management capability for improving logistics performance. It was shown that the demand management capability of logistics services effectively coordinates efforts and integrates capabilities in company to improve performance, providing essential evidence and information to support operational managers. In this way, the study helps companies identify the key LCFs that influence their performance and prioritize initiatives to improve operational efficiency. This approach ultimately contributes to the development of a more efficient and effective logistics system that meets customer needs while optimizing costs and increasing competitiveness in the marketplace.

This study is based on the application of experts’ knowledge using ISM-MICMAC. It has certain limitations and also presents opportunities for future research as well. First, the study involves only a limited number of experts in the evaluation process. However, the framework developed in this study could be used in the future to explore the problem more comprehensively with a larger pool of experts. Future research could include additional capability factors from different subsectors of the SC and construct models using modified ISM (MISM) or total ISM (TISM) in conjunction with MICMAC. In addition, research could focus on establishing structural relationships among the capability factors by using SEM, network analysis (NA), or Bayesian networks (BN). Our follow-up research, based on a mixed-methods approach, will apply DEMATEL-ISM-MICMAC to investigate the cause-and-effect relationships of logistics capability factors and to rank them in order of preference.

## CRediT authorship contribution statement

**Mohammad Kamrul Hasan:** Writing – review & editing, Writing – original draft, Visualization, Validation, Software, Resources, Methodology, Investigation, Funding acquisition, Formal analysis, Data curation, Conceptualization. **Xunping Lei:** Writing – review & editing, Visualization, Validation, Software, Methodology, Investigation, Funding acquisition, Formal analysis, Data curation, Conceptualization. **Arbia Hlali:** Writing – review & editing. **Zixiang Bian:** Writing – review & editing, Writing – original draft, Visualization, Validation, Software, Methodology, Formal analysis, Data curation, Conceptualization.

## Statement on informed consent

The informed consent is taken from the participant.

## Data availability

Data included in article.

## Funding

This research is financially supported by the Natural Science Foundation General Project of Anhui Province, China, [Grant NO. 2108085MG246] and Research on Digital Economy Driving High-quality Development of Yangtze River Delta Logistics Industry, [Grant NO. 2023AH051641]

## Declaration of competing interest

The authors declare that they have no known competing financial interests or personal relationships that could have appeared to influence the work reported in this paper.
